# Familial multiple sclerosis and association with other autoimmune diseases

**DOI:** 10.1002/brb3.899

**Published:** 2017-12-19

**Authors:** Vanesa Pytel, Jordi A. Matías‐Guiu, Laura Torre‐Fuentes, Paloma Montero, Álvaro Gómez‐Graña, Rocío García‐Ramos, Teresa Moreno‐Ramos, Celia Oreja‐Guevara, Miguel Fernández‐Arquero, Ulises Gómez‐Pinedo, Jorge Matías‐Guiu

**Affiliations:** ^1^ Department of Neurology Institute of Neurosciences Hospital Clínico San Carlos Madrid Spain; ^2^ Neurobiology Laboratory Institute of Neurosciences Hospital Clínico San Carlos Madrid Spain; ^3^ Department of Immunology Hospital Clínico San Carlos Instituto de Investigación Sanitaria San Carlos Universidad Complutense de Madrid Madrid Spain

**Keywords:** autoimmune diseases, familial multiple sclerosis, inheritance, multiple sclerosis

## Abstract

**Objectives:**

Autoimmune diseases (AID) follow a complex, probably polygenic, pattern of inheritance and often cluster in families of patients with multiple sclerosis (MS). Our objective was to analyze family patterns and characteristics in families including more than one patient with MS.

**Materials and Methods:**

We analyzed personal and family history of neurological, systemic, and autoimmune diseases in 84 MS patients from 40 different families. Families were classified in two groups: families with cases of MS in at least two different generations (15 families) and families in which cases of MS belonged to only one generation (25 families).

**Results:**

The two previously established groups presented different clinical patterns and frequency of association with another AID. In one group, the second generation displayed a higher annual relapse rate than the first generation, higher frequency of progressive forms of MS, and more patients with another AID in addition to MS. Relapsing‐remitting forms of MS (RRMS) were more frequent in the other group.

**Conclusions:**

Families that include more than one MS patient may show two distinct patterns. This finding seems important for the compression and analysis of genetic information on MS.

## 1 INTRODUCTION

Autoimmune diseases (AID) result from an impaired immune system response, probably arising from an interaction between multiple genetic and environmental factors. Given that several types of AID are often present in the same person or family, they may have shared pathophysiological mechanisms. AID are more frequent in patients with nonfamilial multiple sclerosis (MS) than in the general population (Dobson & Giovannoni, 2013).

AID follow a complex, probably polygenic, inheritance pattern; occurrence of these diseases in different families is therefore heterogeneous. Associations between different AID in the same family may be explained by several mechanisms. On the one hand, co‐occurrence of several AID types may be promoted by certain genetically predetermined mechanisms combined with other genetic and/or environmental factors associated with each specific disease. The occurrence of multiple autoimmune diseases in a family, rather than in a single individual, may support this hypothesis. On the other hand, certain genetic factors are thought to confer a predisposition to specific diseases that may share pathophysiological mechanisms (Goris & Liston, 2012). Copresence of several AID types in a single individual supports this second hypothesis (Lorber, Gershwin, & Shoenfeld, 1994).

Evidence shows that the interaction of numerous genetic and environmental factors contributes to the development of MS (Esposito et al., 2015). The impact of genetic factors is clearly seen in monozygotic and dizygotic twins, who display different disease concordance rates. In family clusters, the incidence of the disease is higher in siblings and in close relatives of the proband; according to several family studies, 15% to 20% of the patients with MS have a relative with the disease. Studying inheritance patterns in MS and other types of AID may improve our understanding of the interactions between genetic and environmental factors. Therefore, examining the clinical profiles of families exhibiting cases of MS and an additional AID may help us determine the association between MS and other AID types. The aim of our study was to analyze clinical profiles, inheritance patterns, and associations with another AID in families with more than one member affected by MS.

## 2 MATERIALS AND METHODS

The study included 84 patients with a diagnosis of MS and at least one first‐ or second‐degree relative also diagnosed with MS; patients represented a total of 40 families. All participants met the 2010 McDonald criteria (Polman et al., 2011), which were also used to classify according to the form of MS: relapsing‐remitting (RRMS), primary progressive (PPMS), and secondary progressive (SPMS).

To avoid selection, recall, and information biases, and taking into account the fact that MS incidence in families varies depending on the degree of kinship and decreases significantly with genetic distance from MS proband (Kalman & Leist, 2004; Nielsen et al., 2005; O'Gorman, Lin, Stankovich, & Broadley, 2003), our analysis included only two generations. However, the total AID count for each family did include more distant relatives with AID; these data were reported by probands themselves after indirect questioning (Figure S1).

We compiled the patients’ personal and family histories of neurological, systemic, and autoimmune diseases using a questionnaire specifically designed for this purpose. To this end, we used a modified version of the list of autoimmune and autoimmune‐related diseases created by the American Autoimmune Related Diseases Association (2016) (Table S1). The majority of the MS patients, and at least one MS patient from each family, were followed up at our center. The remaining patients were contacted and evaluated in person or by telephone, using the information available in the databases of the Community of Madrid with their authorization. Similarly, non‐MS individuals from the families were contacted and evaluated. All subjects were recruited between March and November 2016. In one family, only the proband could be contacted; this individual was indirectly questioned about other family members.

The following clinical and demographic characteristics were recorded: sex, age, age at disease onset (ADO; defined as age of presentation of the first neurological symptom associated with MS), time of duration of disease (TDD; defined as years from the presentation of the first neurological symptom associated with MS to the date of inclusion in the study), clinical form, and annual relapse rate (ARR).

Data were analyzed using IBM^®^ SPSS statistical software, version 20. The Shapiro–Wilk test was used to determine whether variables followed a normal distribution. The descriptive analysis was performed using either absolute frequencies and percentages (n [%]) or mean ± SD for quantitative variables following a normal distribution or as medians (IQR) when distributions were not normal. Given the sample size, nonparametric tests were used for the comparison of clinical and demographic characteristics between groups. The chi‐square test was used to compare independent samples with qualitative variables and the Mann–Whitney U test for continuous variables. Intergroup differences were analyzed using the Kruskal–Wallis H test and Dunn's post hoc test. Statistical significance was set at *p* < .05.

This study complies with the ethical standards of the research committee at our center and the 1964 Declaration of Helsinki and its subsequent amendments.

## 3 RESULTS

We included 40 families in which more than one member had MS; the total sample consisted of 84 patients (33 men, 39.30%; 51 women, 60.70%). Pedigrees are shown in Figure S1. Mean age in the sample was 46.59 ± 11.83 years (range, 23–79). Fifty‐three patients (63.10%) belonged to families with only one affected generation (siblings and/or first cousins), whereas the remaining 31 (36.90%) belonged to families with two or more affected generations. RRMS was found in 56 patients (66.70%), PPMS in nine (10.70%), and SPMS in 19 (22.60%). Mean ADO was 28.72 ± 9.39 years (range, 14–55) and mean TDD was 17.63 ± 10.92 years (range, 1.64–56.06). The mean ARR was 0.40 ± 0.35 (Table S2). MS was associated with at least one other AID in 20 patients (23.80%). Excluding patients with MS and the coexistence of another autoimmune disease, 38 patients with multiple sclerosis in our population (45.23% of the total sample) had at least one other family member with another autoimmune disease (only one relative in 17 [20.23%]; more than one relative in 21 [25%]). Nineteen families (47.50%) had members with an AID other than MS; 23 (57.50%) had members with both MS and another AID. The median number of cases per family of an AID other than MS (including members with both MS and another AID) was 1 (0–2.75).

**Figure 1:**
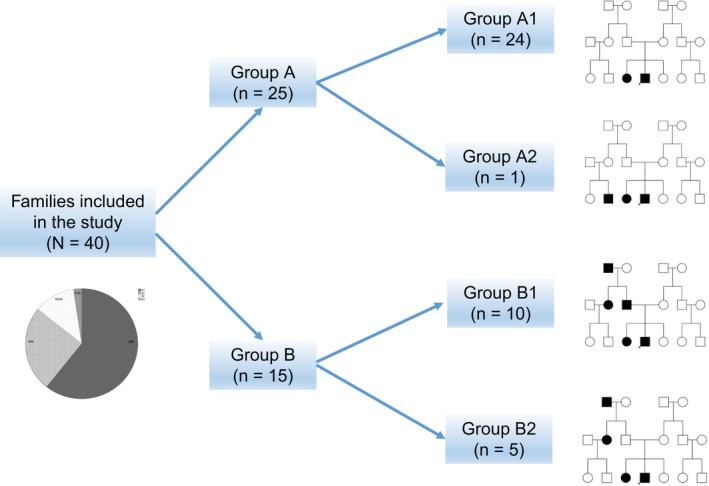
Graph representing the 40 families included in the study. They were initially classified into two groups: group A (families in which MS‐affected patients belong to the same generation; *n *= 25) and group B (families in which MS‐affected patients belong to two or more generations; *n *= 15). These groups were further classified into subgroups A1 (affected siblings only; *n *= 24), A2 (affected siblings and cousins of the same generation; *n *= 1), B1 (affected parents and offspring with or without affected uncles/aunts or grandparents; *n *= 10), and B2 (affected siblings and uncles/aunts and parents without MS;* n *= 5)

The families included in this study were initially classified into groups A, in which all patients with MS belonged to the same generation (25 families, 62.5%), and B, in which more than one generation was affected by MS (15 families, 37.5%). We further subdivided families according to the degree of kinship between the affected members: A1 included 24 families (60%) where affected members were siblings (51 cases, 60.71%) and A2 included one family (2.5%) in which MS patients included siblings and cousins of the same generation (two, 2.38%); B1 included 10 families (25%) in which the affected members were parents and offspring, either with or without affected uncles/aunts or grandparents (21 cases, 25%) and B2 included five families (12.5%) in which the proband, siblings, and uncles/aunts had MS but the parents did not (10 cases, 11.90%) (Figure 1).

The median number of affected patients per family was greater in group B (3 (2,3)) than in A (2 (2‐2)) (*p* = .05). Of the total sample, 53 patients (63.10%) were in group A and 31 (36.90%) were in B. Group A included 32 women (60.40%) and 21 men (39.60%), and B had 19 women (61.30%) and 12 men (38.70%). Mean ADO was 28.69 ± 8.81 years (range, 14–48) and 28.77 ± 10 years (range, 14–55), respectively (*p* = .85). RRMS was more frequent in A (41 cases, 77.40%) than in B (15 cases, 48.40%). Conversely, progressive forms (PF: PPMS and SPMS) were more prevalent in B (16 cases, 51.60%) than in A (12 cases, 22.60%) (*p* < .01). Compared to A, B included significantly more patients with both MS and another AID (13 cases [41.90%] vs. seven [13.20%]; *p* < .01). In the subsequent analysis, we excluded A2 because the group contained only one family. B2 had significantly more affected members per family than did other groups (B2, 3 ± 0.00; A1, 2.12 ± 3.33; B1, 2.30 ± 0.48; *p* < .01).

Likewise, A1 and B2 displayed significantly more cases of RRMS than of PF (RRMS: A1, 40 cases [78.40%]; B1, seven [33.33%]; B2, eight [80%]; PF: A1, 11 [21.60%]; B1, 14 [66.70%]; B2, two [20%]; *p* < .01). Compared to A1, groups B1 and B2 included a greater proportion of patients with both MS and another AID (A1, seven cases [13.7%]; B1, nine [42.9%]; B2, four [40%]; *p* = .01) (Table 1).

**Table 1:** Characteristics of families and MS‐affected patients classified by subgroup. A comparative analysis of the different subgroups was performed (A2 was excluded from the analysis because it only comprised one family)

	A1*n *= 24 families*n *= 51 patients	A2*n *= 1 family*n *= 2 patients	B1*n *= 10 families*n *= 21 patients	B2*n *= 5 families*n *= 10 patients
Sex, female	32 (62.70%)	0 (0%)	14 (66.70%)	5 (50%)
Age at disease onset, mea*n *± SD (range)	29.16 ± 8.65 (14–48)	17 ± 2.82 (15–19)	30.19 ± 10.99 (14–55)	25.80 ± 8.94 (15–46)
Number of MS patients per family^a^	2 (2–2)	3 (0–0)	2 (2–3)	3 (3–3)
Clinical form of MS (RRMS)^a^	40 (78.40%)	1 (50%)	7 (33.33)	8 (80%)
ARR, mean ± SD (range)	0.42 ± 0.31 (0–1.49)	0.25	0.36 ± 0.33 (0–1.22)	0.45 ± 0.58 (0–1.96)
Patients with MS + another AID^a^	7 (13.70%)	0 (0%)	9 (42.9%)	4 (40%)
Patients with AID and no MS per family, median (IQR)	0 (2–0)	0 (0–0)	1 (0–1.25)	0 (0–1.5)

a *p* ≤ .05.

In B, MS was inherited from the father in six families (40%) and from the mother in nine (60%). Paternal transmission was most frequently associated with RRMS (eight cases, 80%). PF were less frequent (PPMS, two cases [20%]; SPMS, 0 [0%]). Maternal transmission, on the other hand, was linked to RRMS (six cases, 46.2%) and SPMS (seven, 53.80%); no cases of PPMS were found (*p* = .01). Families with paternal transmission had a median of 3 (Goris & Liston, 2012; Lorber et al., 1994) affected members per family, whereas those with maternal transmission had 2 (2–3); differences were not statistically significant. No significant differences were found between patients with maternal and those with paternal transmission in terms of ADO and ARR. A trend toward a higher frequency of maternal transmission in B1 (eight cases, 80%) than in B2 (one, 20%) was observed (*p* = .08). The single family included in A2 showed paternal transmission and had three affected members (two with RRMS and one with PPMS).

Comparison of the two generations of B1 revealed that the disease presented earlier in offspring than in parents (mean ADO: 23.73 ± 6.42 years in offspring vs. 37.30 ± 10.76 in parents; *p* < .01), which is suggestive of an anticipation phenomenon (AP). Likewise, the second generation displayed a higher ARR than the previous one (0.54 ± 0.36 vs. 0.16 ± 0.15; *p* = .01).

Twenty patients had both MS and another AID; 13 were women (65%) and seven were men (35%). Of these, seven (35%) were in A and 13 (65%) in B. No patient had more than one AID in addition to MS. The most frequent types of AID were autoimmune thyroiditis (25%), type 1 diabetes mellitus (20%), and autoimmune uveitis (15%). In 16 families (40%), at least one patient had both MS and another AID (Table 1).

Regarding the temporal connection between AID and MS, the other AID presented after the onset of MS in eight patients (40%) and before MS in 10 patients (50%); this variable was unknown in two cases. In only one case (5%), the onset of the other AID, systemic lupus erythematosus (SLE), was associated with a change in MS treatment.

Family history of non‐MS AID was higher in those patients with another AID (15 of 20 [75%] vs. five of 20 [25%], *p *< .01). No differences in the temporal connection between ADO and onset of the other AID were observed between patients with and without a family history of AID. Copresence of MS and another AID was more frequent in B (13 cases, 41.90%) than in A (seven cases, 13.20%) (*p* < .01).

AID other than MS was present in 45.23% of the patients. The most frequent AID in relatives with no MS was autoimmune thyroiditis (45%). Table 1 shows the frequency of non‐MS AID by family for each subgroup. Although presence of other AID was more frequent in A1 and B1, differences between A1, B1, and B2 were not significant.

Table 2 compares the presence of another AID in the families between A and B. Including cases of MS plus another AID, around 57.50% of the families had a case of non‐MS AID. Excluding these cases, the percentage decreases to 47.50%. Furthermore, 30% of the families belonging to A and 27.50% of those belonging to B had a member with a non‐MS AID. There were no differences between families with MS in one or more than one generation. Table 3 compares patients’ profiles according to whether their families included members with other AID. In these families, MS most frequently appeared in the form of RRMS.

**Table 2:** Presence of other AID by group. Group B contained more families including individuals with MS plus other AID than group A

Families (*n *= 40)	Families including individuals with MS plus other AID			Families including individuals with other AID (co‐occurrence and/or family history)			Families including individuals with other AID (no MS)			Families including individuals without other AID		
	Total	Group A	Group B	Total	Group A	Group B	Total	Group A	Group B	Total	Group A	Group B
Total families	16 (40%)	6 (15%)^a^	10 (25%)^a^	23 (57.50%)	12 (30%)	11 (27.50%)	19 (47.5)	11 (27.50%)	8 (20%)	17 (42.50)	13 (32.50%)	4 (10%)
Patients with other AID per family	2 (2–3.75)	2.50 (1.75–4)	2 (1.75–3.25)	2 (1–3)	2.50 (1.25–3.75)	2 (1–3)	2 (1–3)	2 (1–3)	1 (1–2)	0	0	0
Patients with other AID per family (2 generations)	2 (1.25–3)	2.50 (1.75–4)	2 (1–3)	2 (1–3)	2.50 (1.25–3.75)	2 (1–3)	2 (2–3)	3 (2–4)	2 (1.25–3)	0	0	0

a Bold *p* ≤ .05.

**Table 3:** Patient profiles in families with and without other AID. We analyzed patient characteristics by presence/absence of other AID (families including members with MS plus other AID, families with other AID, families without other AID) and by family groups (A or B). Percentages are calculated out of the total number of MS patients in each family category. Group A included more patients with RRMS than group B did in families with other AID (including MS + other AID)

Cases (*n *= 84)	Families including individuals with MS plus other AID			Families including individuals with other AID (co‐occurrence and/or family history)			Families including individuals with other AID (no MS)			Families including individuals without other AID		
	Total	Group A	Group B	Total	Group A	Group B	Total	Group A	Group B	Total	Group A	Group B
Sex, female	18 (54.50%)	7 (21.20%)	11 (33.30%)	28 (59.60%)	16 (34.00%)	12 (25.50%)	24 (61.50%)	15 (38.50%)	9 (23.10%)	23 (62.20%)	16 (43.20%)	7 (18.90%)
Age at disease onset, mean ± SD	29.88 ± 8.99	31.31 ± 8.26	28.95 ± 9.53	29.45 ± 9.60	29.24 ± 8.53	29.68 ± 10.89	29.59 ± 9.52	29.00 ± 8.09	30.44 ± 11.50	27.74 ± 9.16	28.15 ± 9.21	26.56 ± 9.44
Clinical form of MS (RRMS)	22 (66.70%)	10 (30.30%)	12 (36.40%)	33 (70.20%)	**21 (44.70%)** a	**12 (25.50%)** a	28 (71.80%)	19 (48.70%)	9 (23.10%)	23 (62.20%)	20 (54.10%)	3 (8.10%)
ARR, mean ± SD	0.47 ± 0.46	0.57 ± 0.45	0.40 ± 0.46	0.44 ± 0.40	0.49 ± 0.38	0.39 ± 0.44	0.47 ± 0.43	0.49 ± 0.39	0.45 ± 0.50	0.34 ± 0.26	0.33 ± 0.20	0.37 ± 0.40

a Bold *p* ≤ .05.

## 4 DISCUSSION

MS is an autoimmune and probably polygenic disorder resulting from the interaction of multiple genetic and environmental factors (Villar‐Quiles et al., 2016). We classified all patients with familial MS and analyzed the copresence of other types of AID based on the hypothesis that they may share some genetic and environmental factors with MS. To this end, we initially established two groups depending on whether MS was present in a single generation (A) or in two or more generations (B), then further subdivided these groups by degree of kinship between the affected patients (A1, A2, B1, and B2).

In line with our hypothesis, we identified differences: compared to families with two generations affected by MS, the families with MS in only one generation displayed a significantly higher frequency of RRMS (as has previously been reported in the literature (Fernández‐Pérez et al., 1999)), a lower number of affected members, and fewer cases of MS associated with another AID. Prevalence of RRMS was greater in families in which the generation preceding the proband had no cases of MS (A and B2) than in those with two successive generations of MS patients.

Our results do not reflect the Carter effect, that is, higher frequency of paternal transmission of MS; in our sample, both lines of inheritance affected offspring similarly, with maternal transmission being slightly more frequent. In addition to this hypothesis, some researchers (Kantarci et al., 2006) have suggested that paternal transmission may result in more severe forms of MS; in contrast, maternal transmission has been associated with early ADO (Barcellos et al., 2002; Hupperts et al., 2001). In the families in our study, however, maternal transmission was more frequently associated with SPMS than paternal transmission, especially in families with affected members in two successive generations. Despite these findings, our results do not seem to support the idea that the line of inheritance affects clinical presentation.

Families in B1 displayed AP, a biological phenomenon whereby ADO decreases with each successive generation (Friedman, 2011), that is, if offspring of affected parents develop the disease, symptoms will become apparent at earlier ages. AP is well documented in several neurological disorders (Lemos et al., 2014; Penrose, 1948; Ranen et al., 1995; Rosenmann et al., 1999). Furthermore, we found a significantly higher ARR in the second generation (AP is also associated with increased severity in successive generations). AP is attributed to a number of genetic mechanisms, including expansion of an unstable nucleotide repeat, as in Huntington disease and myotonic dystrophy type 1, and telomere shortening, as in dyskeratosis congenita (Vulliamy et al., 2004) and breast cancer (Martinez‐Delgado et al., 2011). Some researchers have suggested that AP is present in familial forms of AID (Fresko et al., 1998; Giardino et al., 2011; McDermott, Khan, & Deighton, 1996; Picco, Goodman, Reed, & Bayless, 2001). This phenomenon has also been described in familial optic neuromyelitis (Kavoussi & Lesser, 2015) and in various series of patients with MS (Papais‐Alvarenga et al., 2015; Romero‐Pinel et al., 2010). While AP in MS cannot be explained by known mechanisms, it might be hypothesized that it is related to epigenetic factors (Nilbert, Timshel, Bernstein, & Larsen, 2009). In this regard, epigenetic alterations have been reported in MS (Bos et al., 2015).

AID affects 4.5% of the population (2.7% of men and 6. 4% of women), although frequencies vary greatly in female subgroups and prevalence is higher in MS series than in the general population (Hayter & Cook, 2012). Copresence of a non‐MS AID is a factor that can be used to define subgroups in familial forms. The results of a recent meta‐analysis support the hypothesis that AID aggregate in families and have common mechanisms (Cárdenas‐Roldán, Rojas‐Villarraga, & Anaya, 2013). Likewise, a meta‐analysis of the presence of other AID in patients with familial MS demonstrated a higher frequency, among the proband's first‐degree relatives, of autoimmune thyroiditis in particular and of other AID types to a lesser extent; copresence of another AID was less frequent in patients with nonfamilial MS (Dobson & Giovannoni, 2013). Our results show a high frequency of AID in familial MS. At least one other AID had manifested in 23.80% of the patients, meaning that 57.50% of the families had a member with AID, including those with MS plus another AID. The AID most frequently associated with MS was autoimmune thyroiditis. In our sample, the associated AID was equally likely to manifest before or during MS; in only one case did it manifest after a change in immunomodulatory treatment. RRMS was more frequent among patients from families with individuals who had another AID.

Several limitations should be kept in mind when interpreting our results. Firstly, while we analyze the presence of other AID, our study focuses on familial MS and not on AID, so our results apply exclusively to familial MS. Although this approach does not allow us to extrapolate our results to other types of AID, it minimizes the risk of heterogeneity that might have resulted from including probands with any AID. Secondly, including large families may introduce recall biases (Marrie, 2007); we therefore analyzed only two generations, which limited the data we were able to examine, even though we did detect cases in other family branches. Furthermore, we did not perform any complementary tests to detect asymptomatic cases in nonaffected family members, as some authors have recently suggested (Xia et al., 2017). When comparing two generations, we cannot avoid the potential bias associated with treatment, which has varied very significantly in recent decades. Therapeutic differences could influence variations in ARR between generations, although younger generations would be expected to display a lower rate; in group B, the opposite was observed. Several factors may have had an impact on the association between MS and other AID, including time of diagnosis (incidence of each AID may vary with the subject's age) and effect of treatment (several studies have reported onset of a second AID after immunomodulatory therapy in MS, as well as in other types of AID (Perez‐Alvarez, Perez‐de‐Lis, & Ramos‐Casals, 2013)). Lastly, we cannot rule out the potential influence of environmental factors that differ between families, such as the role of potentially modifiable environmental factors, for example vitamin D deficiency.

The need for family studies in genome research in MS is supported by the interesting scientific debate around the most suitable method for detecting genetic forms of the disease. Family studies have been compared to case‐control studies after a genome‐wide association study (GWAS) did not detect an association between MS and a mutation detected in a study of two families with MS. It has been suggested that GWAS is less effective than family studies for detecting rare genomic variants and is more likely to introduce a selection bias because, on occasion, it includes individuals with insufficient neurological assessments as cases and others with a family history of MS as controls (International Multiple Sclerosis Genetics Consortium (IMSGC), 2013) . In any case, analyzing families provides valuable data for genetic research. However, although many series include patients with familial MS, no studies have addressed the mechanisms of inheritance in these particular cases. Our series defines two groups with different profiles. This data may guide future research on genetic factors associated with MS.

## CONFLICT OF INTEREST

The authors have no conflict of interest to declare.

## AUTHORS’ CONTRIBUTIONS

JMG, JAMG, and MFA were involved in the study design; VP, JMG, COG, PM, and RGR evaluated the patients; VP, LTF, and AG were involved in family studies; VP, LTF, AG, and TM coordinated the information; VP, LTF, and JMG contributed to create and management of the database; VP, UGP, JMG, and JAMG performed the statistical analysis; VP, JMG, UGP, JAMG, and MFA analyzed the results; VP, LTF, and JMG contributed to figures and tables; JMG, VP, and JAMG drafted the manuscript; all authors revised and approved the manuscript.
