# Supplementary material for: Familial multiple sclerosis and association with other autoimmune diseases
Source: Brain Behav. 2017 Dec 19;8(1):e00899. doi: 10.1002/brb3.899 (PMC5853641; doi:10.1002/brb3.899)
Supplement: Supplementary file 1 [file BRB3-8-e00899-s001.pdf]

Group A1

3

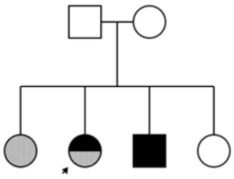

5

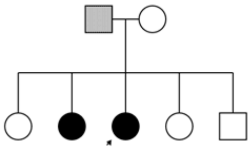

6

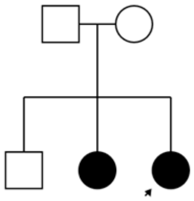

8

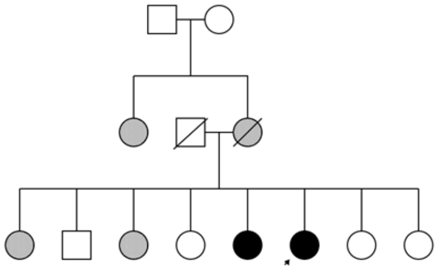

9

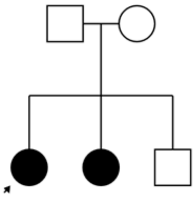

11

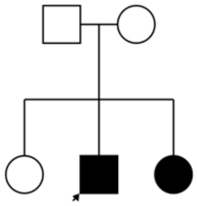

12

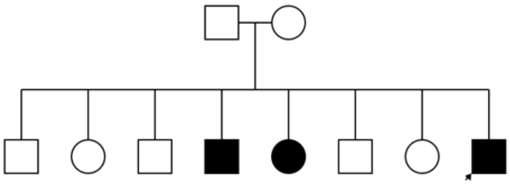

13

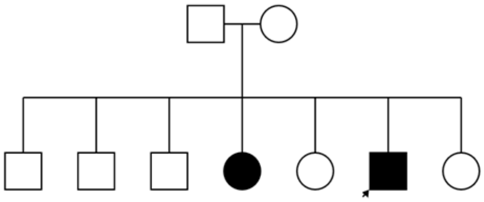

14

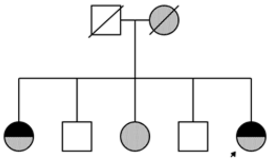

Group A1

15

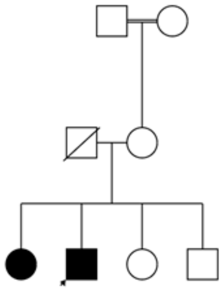

16

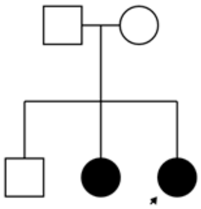

17

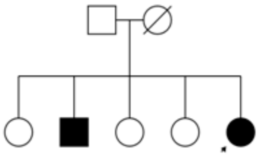

19

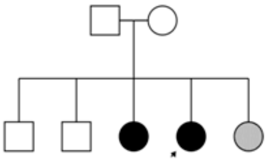

21

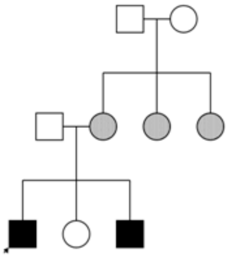

22

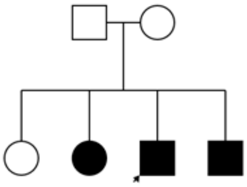

23

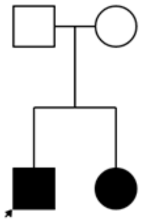

24

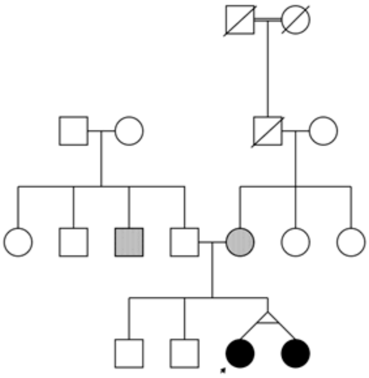

25

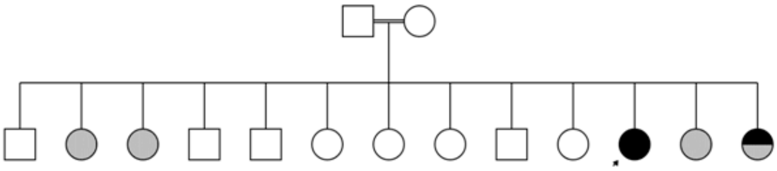

Group A1

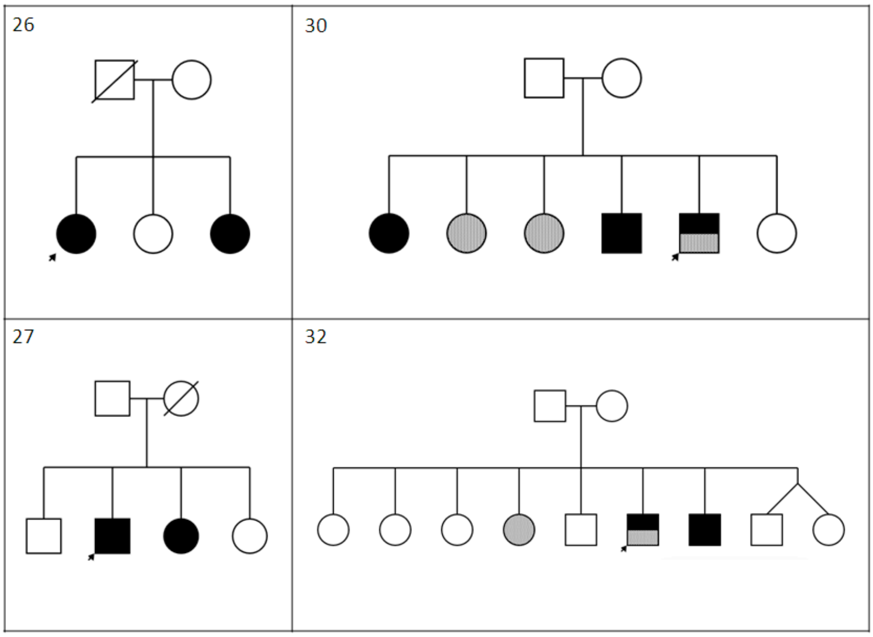

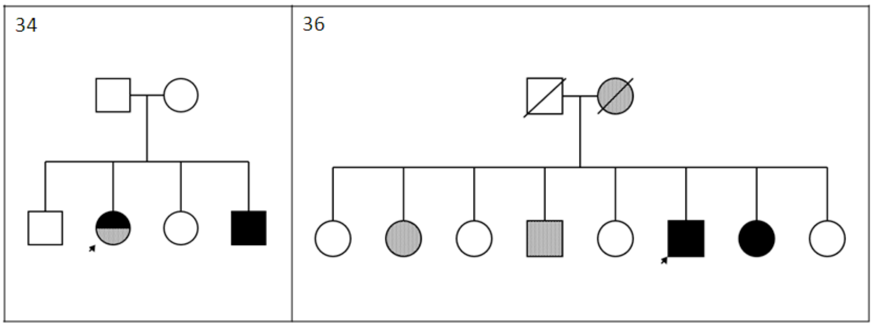

Group A2

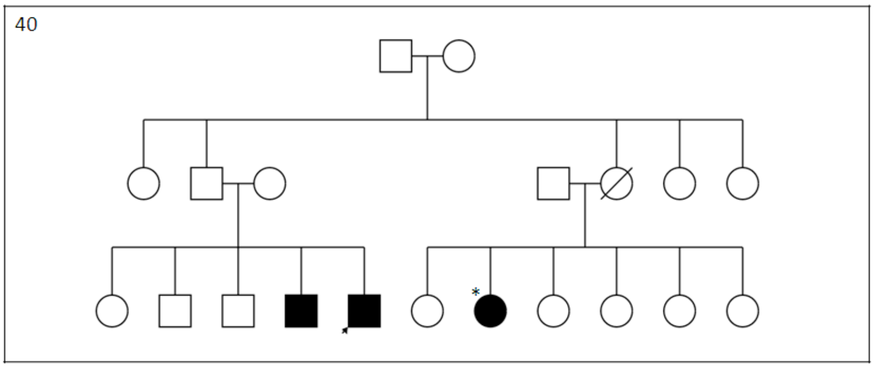

Group B1

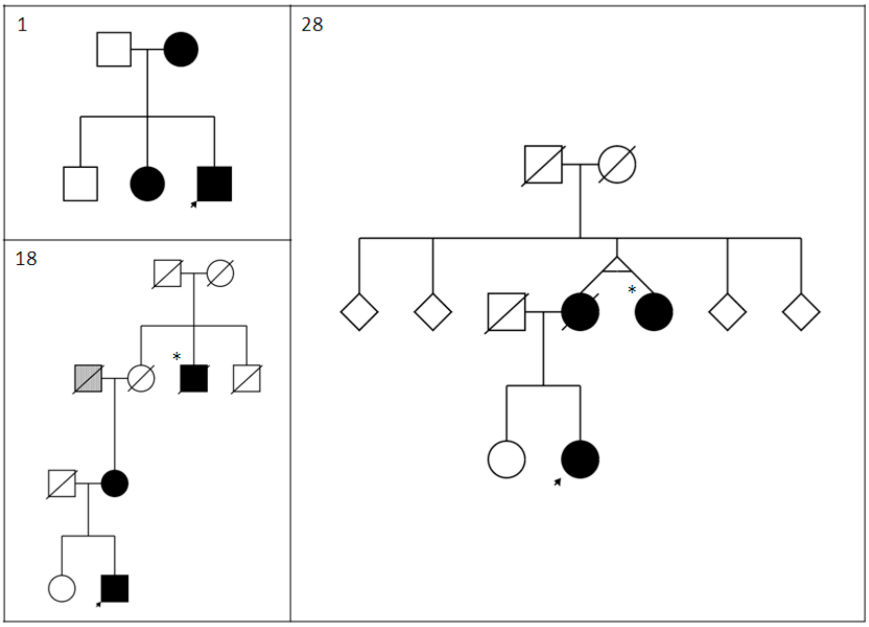

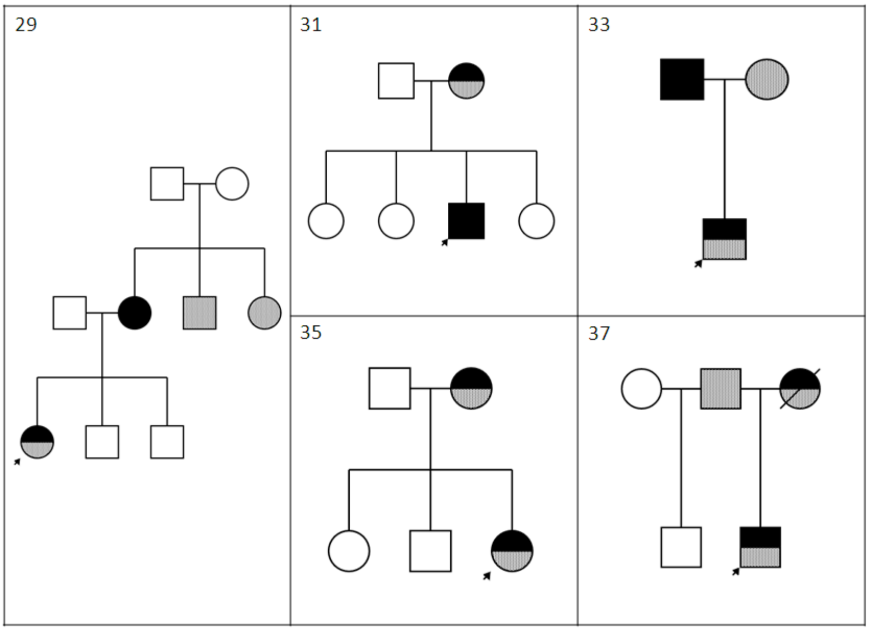

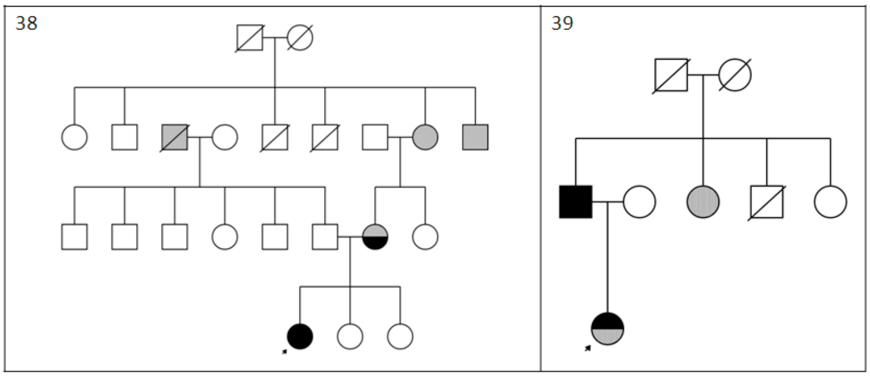

Group B2

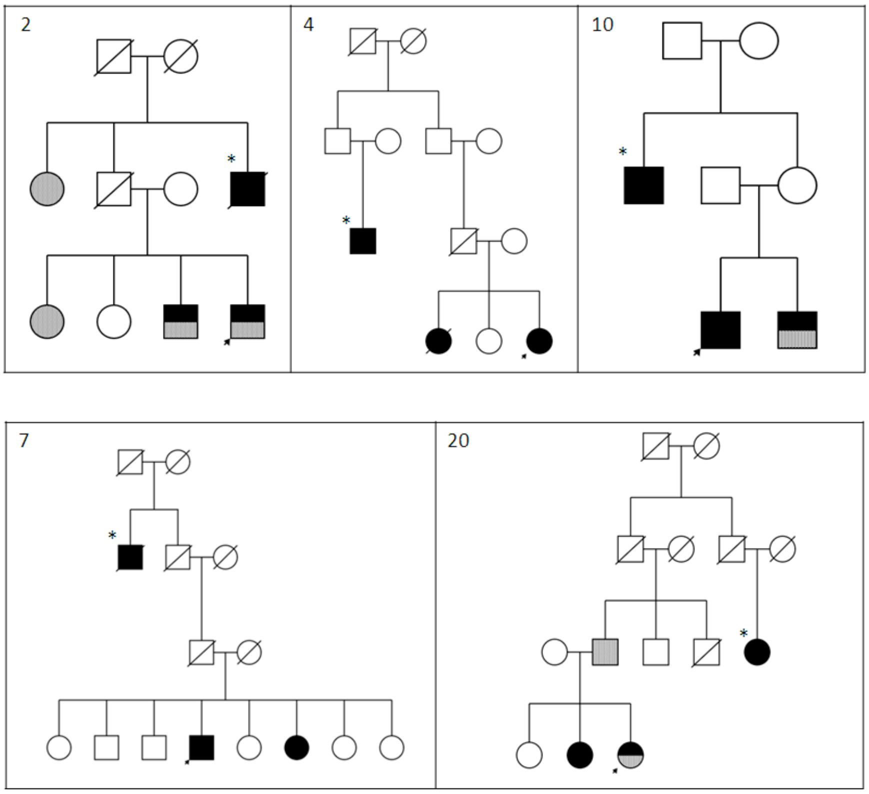

Supporting information, Figure 1. Pedigrees of the 40 families included in the study. Solid black symbol = patient with MS; solid grey symbol = patient with other AID; half black, half grey symbol = patient with MS and another AID; arrow = proband; asterisk = MS patient not included in the study but considered in the calculation of total patients per family (see Material and methods).
