# Supplementary material for: Familial multiple sclerosis and association with other autoimmune diseases
Source: Brain Behav. 2017 Dec 19;8(1):e00899. doi: 10.1002/brb3.899 (PMC5853641; doi:10.1002/brb3.899)
Supplement: Supplementary file 2 [file BRB3-8-e00899-s002.pdf]

Supporting information, Table 1. Modified version of the list of autoimmune and autoimmune-related diseases of the American Autoimmune Related Diseases Association (AARDA, 2016).

**Autoimmune and autoimmune-related diseases**	**Yes/No**
Agammaglobulinaemia	
Rheumatoid arthritis	
Behcet disease	
Ulcerative colitis	
Dermatomyositis	
Juvenile diabetes (type 1 diabetes)	
Celiac disease	
Crohn disease	
Fibromyalgia	
Rheumatic fever	
Glomerulonephritis	
Autoimmune hepatitis	
Lupus (SLE)	
Meniere disease	
Myasthenia gravis	
Narcolepsy	
Neuromyelitis optica (Devic disease)	
Axonal and neuronal neuropathies	
Polyarteritis nodosa	
Polymyalgia rheumatica	
Psoriasis	
Thrombocytopenic purpura	
Sarcoidosis	
Antiphospholipid syndrome (APS)	
POEMS syndrome	
Sjögren syndrome	
Autoimmune thyroid disease	
Uveitis	
Vasculitis	
Vitiligo	
Others (specify)	
