# Supplementary material for: Familial multiple sclerosis and association with other autoimmune diseases
Source: Brain Behav. 2017 Dec 19;8(1):e00899. doi: 10.1002/brb3.899 (PMC5853641; doi:10.1002/brb3.899)
Supplement: Supplementary file 3 [file BRB3-8-e00899-s003.pdf]

Supporting information, Table 2. Description of patients with MS (n = 84). Sex: M/male, F/female. Clinical form: RR/relapsing-remitting, SP/secondary progressive, PP/primary progressive.

	Sex	Age	Subgroup	MS plus other AID	Age at onset	Time since MS onset (years)	Clinical form	Mean ARR
**1**	M	39	B1	No	18	21	SP	0.78
**2**	F	36	B1	No	33	04	RR	1.12
**3**	F	71	B1	No	34	37	PP	0.00
**4**	M	52	B2	Yes	31	21	RR	0.09
**5**	M	54	B2	Yes	46	08	RR	1.96
**6**	F	45	A1	Yes	29	16	RR	0.64
**7**	M	44	A1	No	23	21	RR	0.48
**8**	F	34	B2	No	15	19	RR	0.38
**9**	F	52	B2	No	23	27	PP	0.00
**10**	F	40	A1	No	35	05	RR	0.38
**11**	F	42	A1	No	25	18	RR	0.11
**12**	F	52	A1	No	45	07	RR	0.73
**13**	F	54	A1	No	17	37	SP	Near 0*
**14**	M	53	B2	No	31	22	PP	0.00
**15**	F	51	B2	No	21	30	RR	0.10
**16**	F	46	A1	No	17	29	RR	0.69
**17**	F	47	A1	No	19	28	RR	0.11
**18**	F	47	A1	No	39	08	RR	0.25
**19**	F	45	A1	No	26	19	RR	0.41
**20**	M	37	B2	No	21	16	RR	0.44
**21**	M	30	B2	Yes	21	09	RR	0.46
**22**	M	32	A1	No	23	09	RR	0.45
**23**	F	30	A1	No	20	10	RR	0.21
**24**	M	56	A1	No	20	36	SP	0.34
**25**	F	63	A1	No	43	20	RR	0.10
**26**	M	64	A1	No	28	37	SP	0.41
**27**	M	48	A1	No	40	08	SP	0.25
**28**	F	52	A1	No	40	12	PP	0.00
**29**	F	55	A1	Yes	37	18	RR	0.51
**30**	F	61	A1	Yes	41	20	PP	0.00
**31**	M	46	A1	No	24	23	RR	0.18
**32**	F	48	A1	No	24	24	SP	0.38
**33**	F	44	A1	No	22	22	RR	0.50
**34**	F	45	A1	No	31	14	RR	0.64
**35**	F	46	A1	No	34	12	SP	0.68
**36**	M	57	A1	No	48	09	RR	0.54
**37**	M	39	B1	No	19	20	SP	0.41
**38**	F	70	B1	No	55	15	SP	0.27
**39**	F	42	A1	No	35	07	RR	0.58
**40**	F	44	A1	No	40	04	RR	0.82
**41**	F	40	B2	Yes	30	10	RR	0.73

	Sex	Age	Subgroup	MS plus other AID	Age at onset	Time since MS onset (years)	Clinical form	Mean ARR
**42**	F	34	B2	No	19	15	RR	0.41
**43**	M	42	A1	No	38	04	RR	0.79
**44**	M	34	A1	No	28	07	RR	0.60
**45**	M	33	A1	No	29	04	RR	0.49
**46**	F	36**	A1	No	**	**	RR	**
**47**	M	27**	A1	No	**	**	RR	**
**48**	M	31	A1	No	20	11	RR	0.27
**49**	F	27	A1	No	19	08	RR	0.50
**50**	F	33	A1	No	21	12	RR	0.17
**51**	F	33	A1	No	29	03	RR	0.33
**52**	F	49	A1	No	43	06	RR	1.49
**53**	F	47	A1	Yes	25	22	RR	0.28
**54**	F	49	A1	No	25	24	RR	0.29
**55**	F	52	A1	No	27	25	RR	0.12
**56**	M	48	A1	No	26	22	RR	0.37
**57**	F	46	A1	No	28	19	RR	0.05
**58**	F	49	B1	No	20	29	SP	0.68
**59**	F	77	B1	No	44	31	SP	0.31
**60**	F	50	B1	Yes	32	19	SP	0.27
**61**	F	79	B1	No	23	56	SP	0.07
**62**	M	37	A1	No	35	03	PP	0.00
**63**	F	44	A1	No	34	10	RR	0.31
**64**	M	36	A1	Yes	29	07	RR	1.05
**65**	M	42	B1	No	25	17	SP	0.24
**66**	F	74	B1	Yes	22	52	SP	0.10
**67**	M	45	A1	Yes	22	23	SP	1.32
**68**	M	38	A1	No	25	13	RR	0.46
**69**	M	34	B1	Yes	20	15	RR	0.27
**70**	M	67	B1	No	43	24	SP	0.42
**71**	F	51	A1	Yes	44	07	RR	0.46
**72**	M	42	A1	No	20	22	RR	0.42
**73**	F	36	B1	Yes	31	06	RR	0.18
**74**	F	69	B1	Yes	46	23	SP	0.13
**75**	M	55	A1	No	23	32	SP	0.22
**76**	F	52	A1	No	14	38	RR	0.11
**77**	M	36	B1	Yes	28	08	RR	0.26
**78**	F	59	B1	Yes	32	16	PP	0.00
**79**	F	25	B1	No	14	12	RR	0.52
**80**	F	52	B1	Yes	44	09	RR	0.35
**81**	F	23	B1	Yes	21	02	RR	1.22
**82**	M	57	B1	No	30	27	PP	0.00
**83**	M	39	A2	No	15	24	RR	0.25
**84**	M	41	A2	No	19	23	PP	0.00

* In the last years no relapses have been described.

** These data about patients 46 and 47 were not included in the analyses because this information was obtained indirectly.
